# Time-series forecasting through recurrent topology

**DOI:** 10.1038/s44172-023-00142-8

**Published:** 2024-01-09

**Authors:** Taylor Chomiak, Bin Hu

**Affiliations:** 1 Division of Translational Neuroscience, Department of Clinical Neurosciences, Hotchkiss Brain Institute, Alberta Children’s Hospital Research Institute, Cumming School of Medicine, University of Calgary, Calgary, Alberta T2N 4N1 Canada; 2 Cumming School of Medicine Optogenetics Platform, Hotchkiss Brain Institute, University of Calgary, Calgary, Alberta T2N 4N1 Canada

**Keywords:** Scientific data, Biomedical engineering

## Abstract

Time-series forecasting is a practical goal in many areas of science and engineering. Common approaches for forecasting future events often rely on highly parameterized or black-box models. However, these are associated with a variety of drawbacks including critical model assumptions, uncertainties in their estimated input hyperparameters, and computational cost. All of these can limit model selection and performance. Here, we introduce a learning algorithm that avoids these drawbacks. A variety of data types including chaotic systems, macroeconomic data, wearable sensor recordings, and population dynamics are used to show that *F*orecasting through *Re*current *T*opology (FReT) can generate multi-step-ahead forecasts of unseen data. With no free parameters or even a need for computationally costly hyperparameter optimization procedures in high-dimensional parameter space, the simplicity of FReT offers an attractive alternative to complex models where increased model complexity may limit interpretability/explainability and impose unnecessary system-level computational load and power consumption constraints.

## Abstract (web summary)

Chomiak and Hu introduce a versatile time-series data prediction algorithm using recurring local topological patterning. This algorithm reduces computational complexity and cost, and its feasibility is demonstrated across various dynamic systems including macroeconomic, wearable sensor, and dynamic population systems.

## Introduction

Predicting time-series data has numerous practical applications in many areas of science and engineering as well as for informing decision-making and policy^1–8^. Both the complex and evolving dynamic nature of time-series data make forecasting it among one of the most challenging tasks in machine learning^9^. Being able to decode time-evolving dependencies between data observations in a time-series is critical for interpreting a system’s underling dynamics and for forecasting future dynamic changes^10,11^.

Unlike classical memoryless Markovian processes that assume that an unknown future event depends only on the present state^12^, dynamical systems may retain long-lived memory traces for past system behaviour with respect to its current state^13^. As detecting these traces is particularly challenging for nonlinear systems, we tend to look to increasingly more complex solutions (as a general phenomenon^14^) or even black-box models to decode this type of embedded feature^9,13,15,16^. However, whether increasing model complexity actually increases forecasting performance has been challenged^15^. Moreover, increasing complexity brings with it a variety of drawbacks including various model assumptions, hypothesized parametric equations, and/or vulnerability to overfitting^6,15–22^. There are also often numerous hyperparameters/parameters that require optimization and tuning in high-dimensional parameter space which can have an impact on both a model’s carbon footprint and the cost of machine learning projects^6,15–23^. Complex models have also created another problem; a need to create methods of interpreting/explaining these complex models rather than creating methods that are interpretable/explainable in the first place^24^. In other words, in addition to the interpretable/explainable concerns associated with complex models^24^, there are multiple elements of these models that need to be carefully considered which can limit model selection and performance.

Here, a versatile algorithm for forecasting future dynamic events is introduced that overcomes these drawbacks. Unlike many other algorithms, *F*orecasting through *Re*current *T*opology (FReT) has no free parameters, hyperparameter tuning, or critical model assumptions. It effectively reduces to a straightforward maximization problem with no need for computationally costly optimization and tuning that are required by parameterized models. FReT is based on learning patterns in local topological recurrences embedded in a signal that can be used to generate predictions of a system’s upcoming time-evolution.

## Results

### Proof-of-concept

**Fig. 1:**
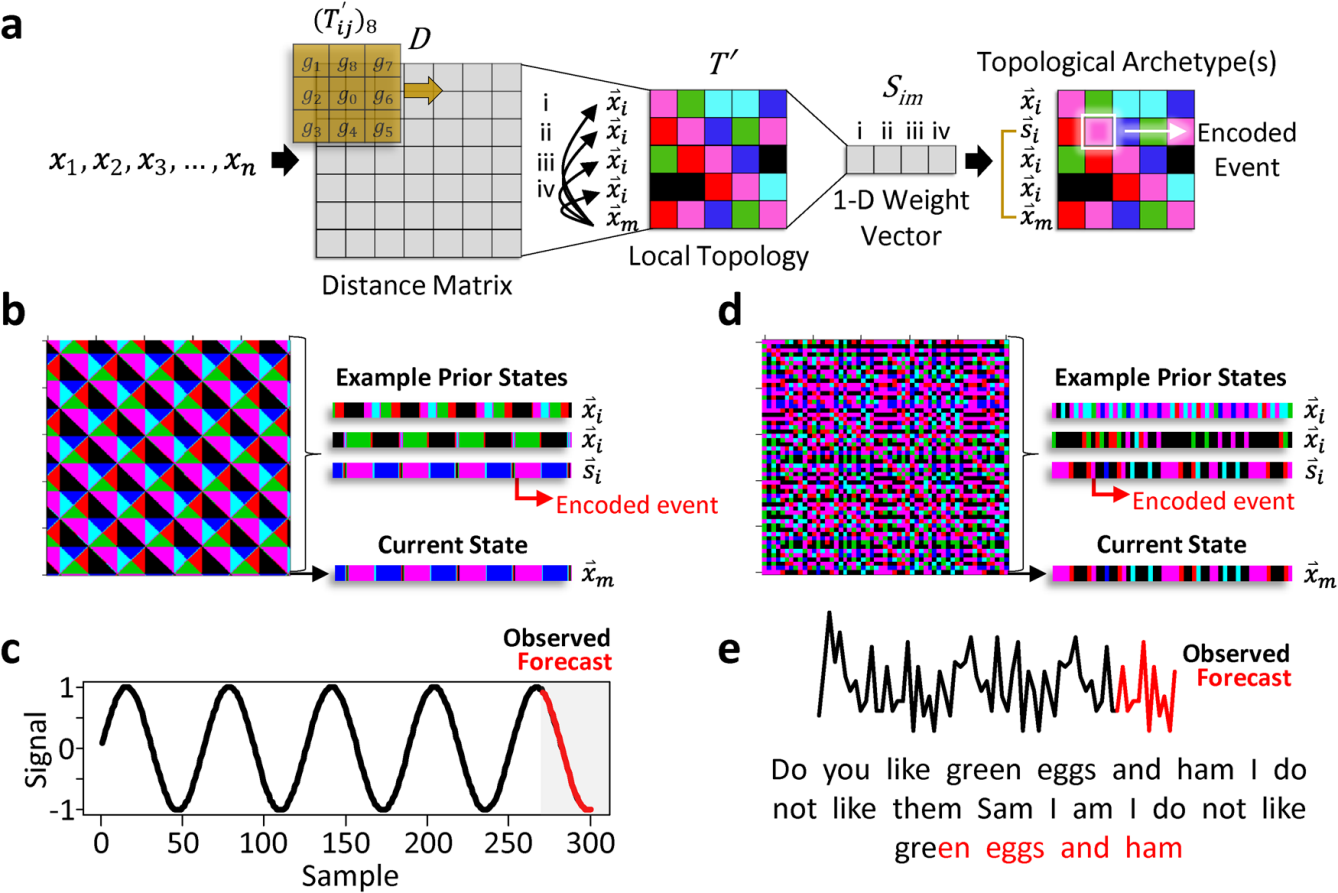
Basic premise of the FReT algorithm. **a** Schematic of the key aspects of the Forecasting through Recurrent Topology (FReT) algorithm. A distance matrix (*D*) is first generated based on an input time-series. Local topology (*T’*) in the form of a flattened 2-D matrix is then extracted from *D* (Eqs. 2–3), which is reduced to a 1-D weight vector (*S*_*im*_) by evaluating ${\mathop{x}\limits^{ \rightharpoonup }}_{m}$ against all ${\mathop{x}\limits^{ \rightharpoonup }}_{i}$ (e.g., i, ii, iii, iv) using Eq. 5. Topological archetype detection (${\mathop{s}\limits^{ \rightharpoonup }}_{i}$) effectively reduces to a simple maximization problem where a set of topological archetypes can be identified from all prior states (${\mathop{x}\limits^{ \rightharpoonup }}_{i}$) that are similar to the system’s current state (${\mathop{x}\limits^{ \rightharpoonup }}_{m}$) (Eq. 6). **b** Embedded patterns in a parametric curve’s (sine wave) surface topology. Different colours signify different local topological neighbourhoods which are coded as integer values 1–6. **c** Once prior states are identified (i.e., archetypes), they can be used to generate an embodied model of unseen future events (e.g., 30-point forecast plotted in red, while the observed data is plotted in black). **d** Identification of encoded events using a well-known Dr. Seuss book excerpt. **e** Using a single topological archetype, the predicted (red) letters for this book excerpt are shown.

FReT works by first constructing a distance matrix based on an input time-series (Fig. 1a). The local topology, in the form of a flattened two-dimensional (2-D) matrix, is extracted from the distance matrix, which is then reduced to a one-dimensional (1-D) weight vector that differentially weights the importance of each part of the input data (Fig. 1a, also see Methods). Each point along the 2-D matrix diagonal represents a point in the signal sequence, and each point gets some context information from every other point in the sequence to capture both long-range and high-level patterns along its associated row vector. The last point in the 2-D matrix diagonal and its associated row vector represent an index of the system’s current state, with all other row vectors representing prior states (Fig. 1b and d). The task is to find the prior state that most closely matches the current state. When formalized in this way, decoding local recurrent topological patterning effectively reduces to a simple maximization problem where a set of topological archetype(s) can be revealed. Once identified, these archetype(s) can be used to create an embodied model of the system’s expected future behaviour, illustrated here with simple sine wave data (Fig. 1c) and a well-known book excerpt: Dr. Seuss’; Do you like green eggs and ham I do not like them Sam I am I do not like green eggs and ham. Here, text data corresponding to the integers 1-26 are used to code the letters a to z (Fig. 1e).

**Fig. 2:**
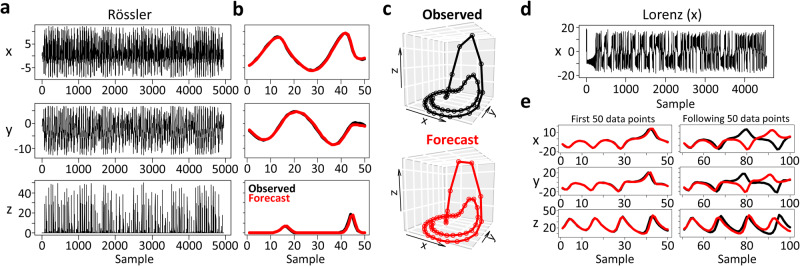
Local topological recurrences and chaotic systems. **a** 3-dimensions of the Rössler attractor system used for archetype identification. **b** Forecasted trajectory for each dimension on unseen data (black: observed ground truth, red: forecast). **c** Multidimensional trajectory of the 50-point Forecasting through Recurrent Topology (FReT) forecast of the unseen test data. **d** The x-dimension of the Lorenz attractor system used for archetype identification. **e** Predicting future events in all x, y, and z dimensions from a single x-dimension archetype across several Lyapunov times. The average normalized root-mean-square-error over approximately one Lyapunov time was close to 11 × 10^−3^, approaching optimized next-generation reservoir computing performance ( ≈ 2-17 × 10^−3^). Panel e, left to right: increasing forecasting inaccuracy with increasing Lyapunov times of the 100-point forecast.

To test whether FReT may represent a method for forecasting upcoming dynamics, it was important to evaluate FReT on more challenging tasks. For this, we first turn to complex dynamic systems as chaotic or complex spatiotemporal behaviours are considered particularly challenging to predict future events^16^. Here, the Rössler attractor system was evaluated as it is often used as a benchmark for testing techniques related to nonlinear time-series analysis^25^ (Fig. 2a). Each signal from this multidimensional attractor was decoded, with the forecasted portion being withheld for testing as the importance of forecasting unseen data cannot be overstated^16^. As shown in Fig. 2b, there was good correspondence between the algorithm’s predicted trajectory and the unseen data, including topological equivalency of the multidimensional signal (Fig. 2c).

Chaotic systems also exhibit patterns of emergent behaviours, i.e., collective patterns and structures which are thought to be unpredictable from the individual components^11^. Thus, whether a single decoded topological archetype could infer unknown future events in unseen dimensions was also tested. For this, another common attractor system was used. Here, only the x-component of the Lorenz attractor system was used to search for a single x-dimension archetype (max(${S}_{{im}}$)) which was then used for predicting future events in all x, y, and z dimensions (Fig. 2d, e). Indeed, it was possible to infer the system’s expected behaviour across all dimensions from decoding the x-dimension component (Fig. 2e, see Supplementary Fig. 1 for Rössler system). This cross-dimensional approach may also help identify similar forecasts with convergent trajectories (Supplementary Fig. 2). For these multi-step-ahead forecasts, the normalized root-mean-square-error was on the order of magnitude of 10^−2^, akin to optimized next-generation reservoir computing (Fig. 2e)^16^. We can also see the characteristic variability in prediction accuracy with increasing forecasting trajectory length^25^ (Fig. 2e).

**Fig. 3:**
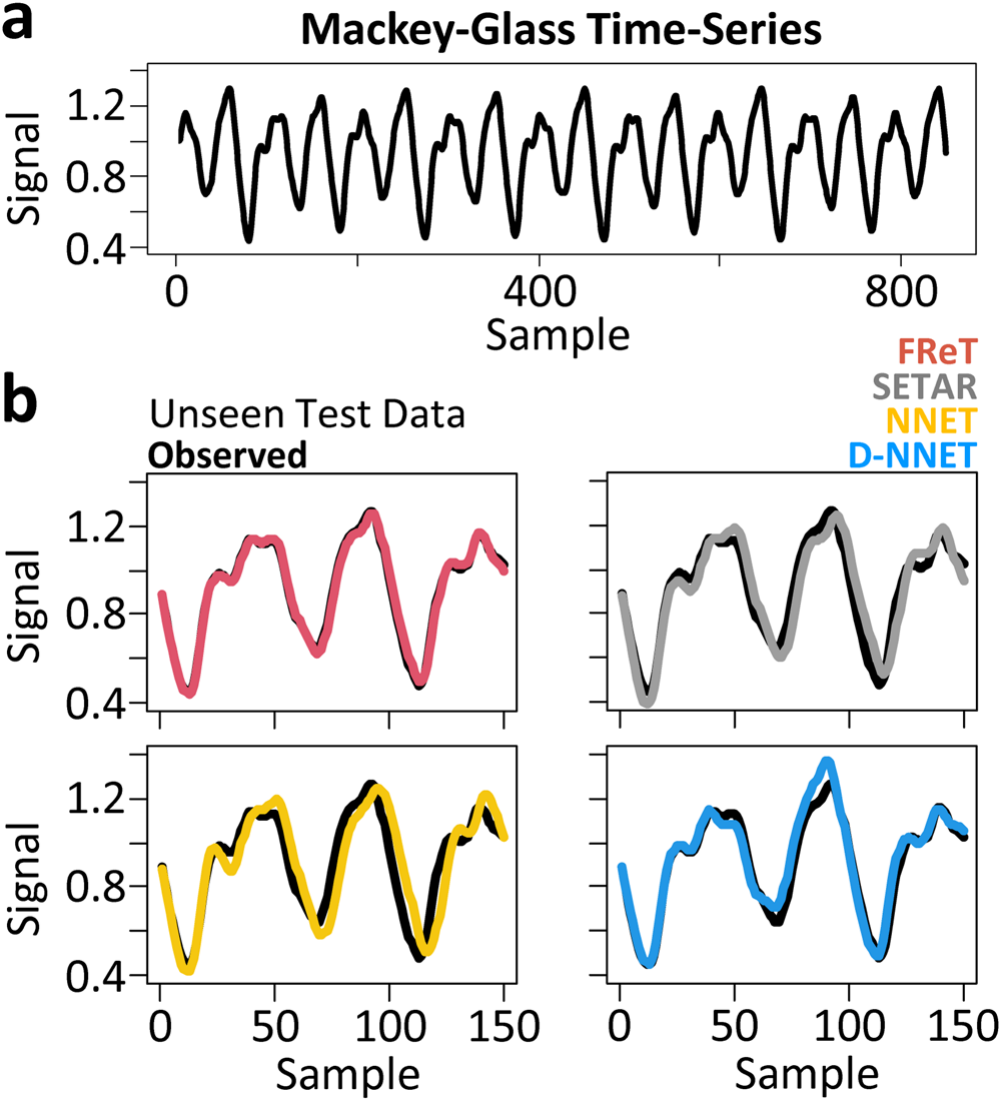
Increasing model parameterization. **a** Mackey-Glass time-series training data. **b** 150-point forecasts of unseen test data with models of increasing parameterization: FReT (red), SETAR (grey), NNET (yellow), and D-NNET (blue). Unseen observed test data shown in black. FReT forecast based on an identified archetype (max(*S*_*im*_)) from each dimension of the Mackey-Glass time-series (cross-dimensional approach). FReT, Forecasting through Recurrent Topology; SETAR, Self-Exciting-Threshold Nonlinear Autoregressive Model; Artificial Neural Network (NNET); Deep-NNET (D-NNET).

To illustrate the efficacy of FReT relative to parameterized models, the multidimensional embedded version of the Mackey-Glass time-series was used next. Mackey-Glass time-series data (Fig. 3a) has real-world relevance as it was initially developed to model physiological control systems in human disease^26,27^. Here, forecasts of unseen test data (Fig. 3b) with commonly used forecasting models of increasing model parameterization were compared: FReT, the self-exciting-threshold nonlinear autoregressive (SETAR) model, an artificial neural network (NNET), and a deep-NNET (D-NNET). SETAR, NNET, and D-NNET hyperparameter optimization and forecast model selection for these data are based on a grid search across 20 embedding dimensions and 15 threshold delays (SETAR) or 15 hidden units (NNET), and 3 layers deep for D-NNET. Variable forecast horizons were also considered, and the root-mean-square-error (RMSE) associated with each model forecast are shown in Table 1. There we can see that a multi-step-ahead FReT forecast was able to outperform these other models for all forecast horizons (Table 1). In fact, despite its simplicity, FReT was also comparable or better than several complex models in a recent study forecasting Mackey-Glass time-series even with a greater forecasting horizon (e.g., FReT 150 step-ahead RMSE = 0.0171 with comparable data normalization)^28^.

**Table 1:** Comparison of root-mean-square-error between models and increasing forecast horizon for the Mackey-Glass dataset.

Model	50 Steps-ahead^a^	100 Steps-ahead^b^	150 Steps-ahead^c^
SETAR Naïve	0.0082	0.0521	0.0652
SETAR Bootstrap	0.0097	0.0575	0.0708
SETAR Block-Boot	0.0093	0.0553	0.0729
SETAR Monte-Carlo	0.0090	0.0513	0.0759
NNET	0.0099	0.0407	0.1048
D-NNET	0.0115	0.0158	0.0472
FReT	0.0054	0.0129	0.0150

FReT, Forecasting through Recurrent Topology; SETAR, Self-Exciting-Threshold Nonlinear Autoregressive Model; Artificial Neural Network (NNET); Deep-NNET (D-NNET). For the SETAR models, different forecasting methods were used for testing including Naïve, Bootstrap resampling, Block-bootstrap resampling, and Monte-Carlo resampling. Grid search across 20 embedding dimensions and 15 threshold delays (SETAR) or 15 hidden units (NNET), and 3 layers deep for D-NNET.

^a^Model hyperparameters for 50-steps-ahead: SETAR mL = 16, mH = 16, thDelay = 14; NNET architecture 17-9-1; D-NNET architecture 7-12-9-4-1.

^b^Model hyperparameters for 100-steps-ahead: SETAR mL = 17, mH = 16, thDelay = 14; NNET architecture 18-8-1; D-NNET architecture 7-14-14-3-1.

^c^Model hyperparameters for 150-steps-ahead: SETAR mL = 15, mH = 16, thDelay = 14; NNET architecture 18-8-1; D-NNET architecture 7-9-13-3-1.

In this section we introduced FReT, an algorithm based on decoding recurrent patterns in a series’ local topology that may offer an effective approach to forecast time-evolving dependencies between data observations in a time-series. To further showcase the versatility of FReT and move beyond idealized systems, several different types of real-world data were tested next that cover different domains and reflect various spatiotemporal evolution patterns.

### Macroeconomic data

**Fig. 4:**
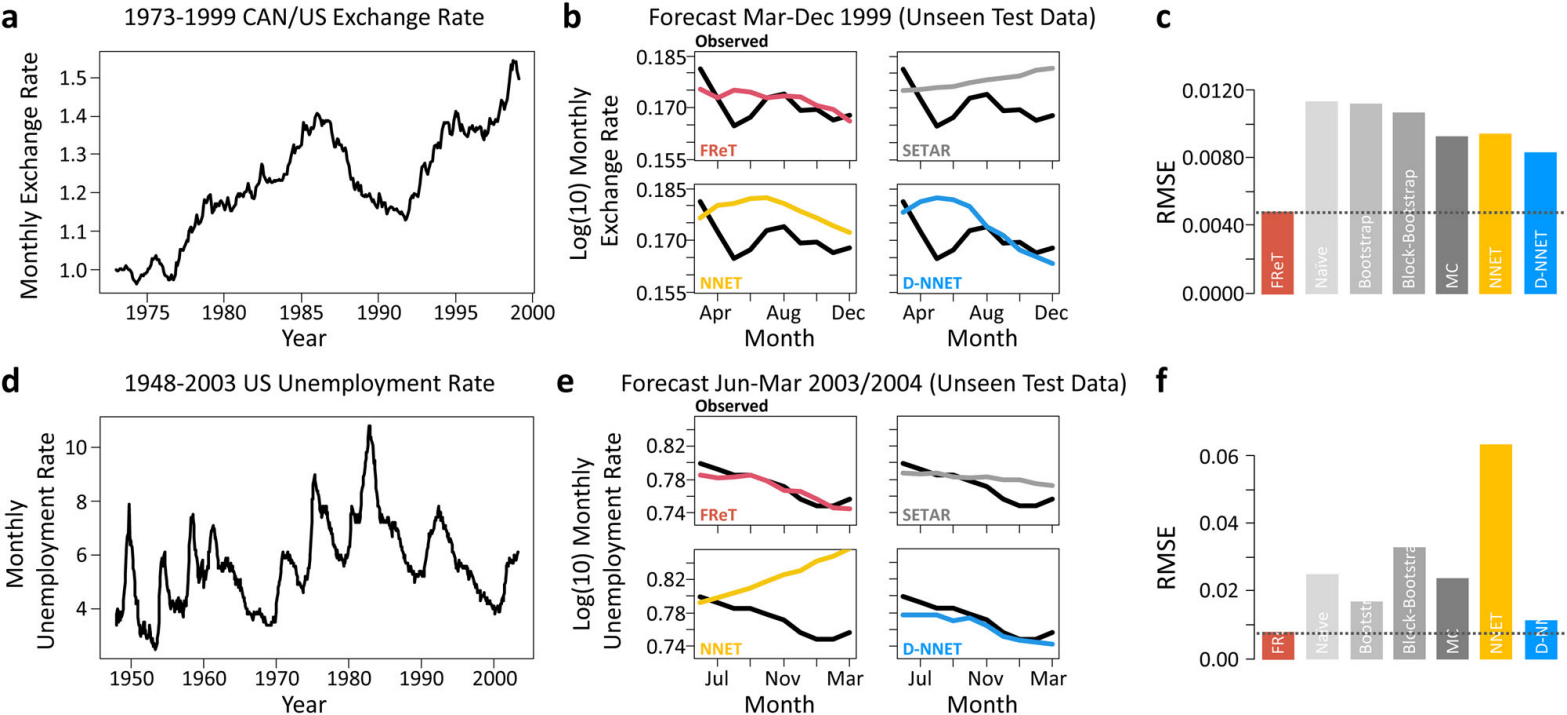
Monthly CAN/U.S. dollar exchange rate and U.S. unemployment rate time-series data. **a** Monthly U.S.-Canada dollar exchange rate from 1973 to 1999. **b** The unseen test data (black) and forecasted exchange rate data for March to December (1999) for the FReT method (red), as well as the SETAR (grey), NNET (yellow), and D-NNET (blue) models. **c** Root-mean-square-error (RMSE) for all models. Model hyperparameters for SETAR: mL = 16, mH = 2, and threshold delay = 14. NNET architecture 14-1-1. D-NNET architecture 8-14-9-4-1. **d** Monthly U.S. unemployment rate from 1948 to 2003. **e** The unseen test data and forecasted unemployment rate data for June 2003 to March 2004 for all models. **f** RMSE for all models. Model hyperparameters for SETAR: mL = 20, mH = 13, and threshold delay = 10. NNET architecture 20-9-1. D-NNET architecture 8-13-13-6-1. All forecasts represent a 10-month forecast. FReT, Forecasting through Recurrent Topology; SETAR, Self-Exciting-Threshold Nonlinear Autoregressive Model; Artificial Neural Network (NNET); Deep-NNET (D-NNET).

The initial set of real-world data that were tested consisted of two well-known and easily accessible macroeconomic datasets available in R. The first dataset represents the monthly U.S. and Canadian dollar exchange rate from 1973-1999 (Fig. 4a, b), while the second dataset reflects the monthly U.S. unemployment rate from 1948-2004 (Fig. 4d, e)^18,29,30^. The last ten months in each dataset were withheld for testing (Fig. 4b and e), and the rest used for training (Fig. 4a and d). To provide additional evidence that FReT can decode important information regarding unseen future events, SETAR, NNET, and D-NNET model data are shown for comparison. While several models performed reasonably well on these data (Fig. 4b and e), FReT was able to reveal some subtle system behaviours regarding the future unseen events (Fig. 4c and f).

### Gait kinematics

**Fig. 5:**
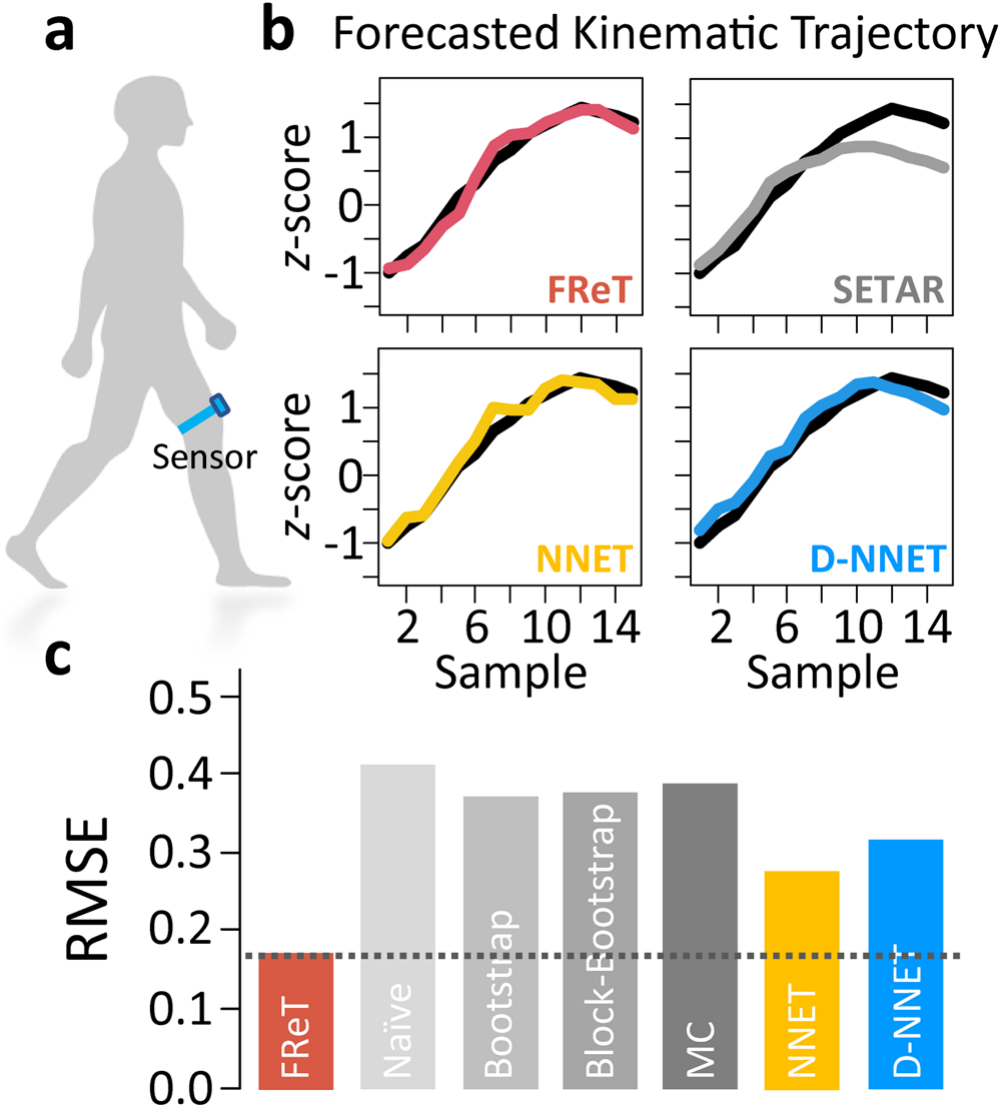
Gait kinematic forecasting with FReT. **a** A schematic of gait kinematic data collection. A single wearable sensor was placed just above the patellofemoral joint line with the use of a high-performance thigh band. **b** An example of FReT (red), SETAR (grey), NNET (yellow), and D-NNET (blue) forecast of unseen test data (black). FReT forecast based on (max(*S*_*im*_)). SETAR model: mL = 2, mH = 6, threshold delay = 4. NNET architecture: 17-2-1. D-NNET architecture: 4-5-3-2-1. **c** Summary (mean) root-mean-square-error (RMSE) data of model forecasts across subjects. FReT, Forecasting through Recurrent Topology; SETAR, Self-Exciting-Threshold Nonlinear Autoregressive Model; Artificial Neural Network (NNET); Deep-NNET (D-NNET).

Given that sensor-aided forecasting of gait kinematic trajectories can improve assistive ambulatory device functionality and user safety^1,31–39^, we next evaluated whether FReT could be used to forecast gait kinematics through the use of wearable motion sensor data. Here, using a single wearable sensor located on the thigh^40^ (Fig. 5a), collected gait data were analyzed. Applying a window of <2 s for input training data (50 data points), FReT was able to outperform SETAR, NNET, and D-NNET in forecasting just over 400 ms of unseen test data even when these models were individually optimized to each individual’s gait (Fig. 5b, c and Supplementary Fig. 3). Moreover, the accuracy of FReT was also comparable or better than recently and independently developed neural network-based models forecasting gait kinematics at half the time horizon (i.e., 200 ms), all of which report average RMSE values based on *z*-score normalized gait-cycle data using wearable sensor data^1,2^.

### Computational Efficiency

**Fig. 6:**
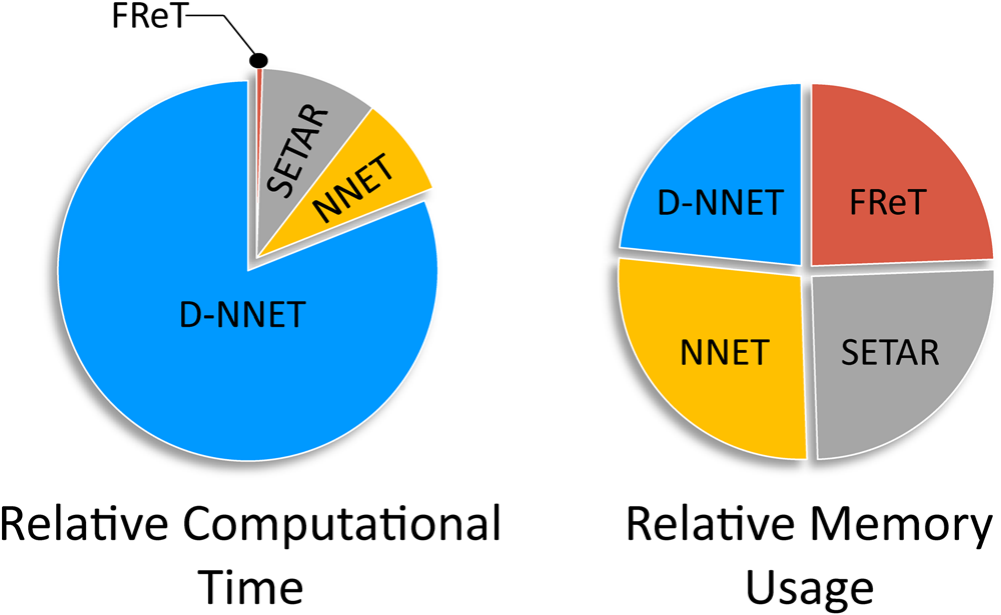
Relative computational time and memory usage across methods. A comparison of the estimated average computational time and memory usage associated with each of the models used.

Although the general effectiveness of complex parameterized models in the prediction of time-series is well-established, there are also often numerous hyperparameters that require optimization and tuning that can limit computational efficiency. To illustrate, estimates of the average computational time and memory usage associated with each of the models used here are shown in Fig. 6. We can see that while on average there is limited advantage in terms of memory usage, FReT does offer a distinct advantage in terms of execution time. This is not surprising given there is no need for hyperparameter optimization with FReT. This represents an important difference between FReT and these other models.

### Population Dynamics

**Fig. 7:**
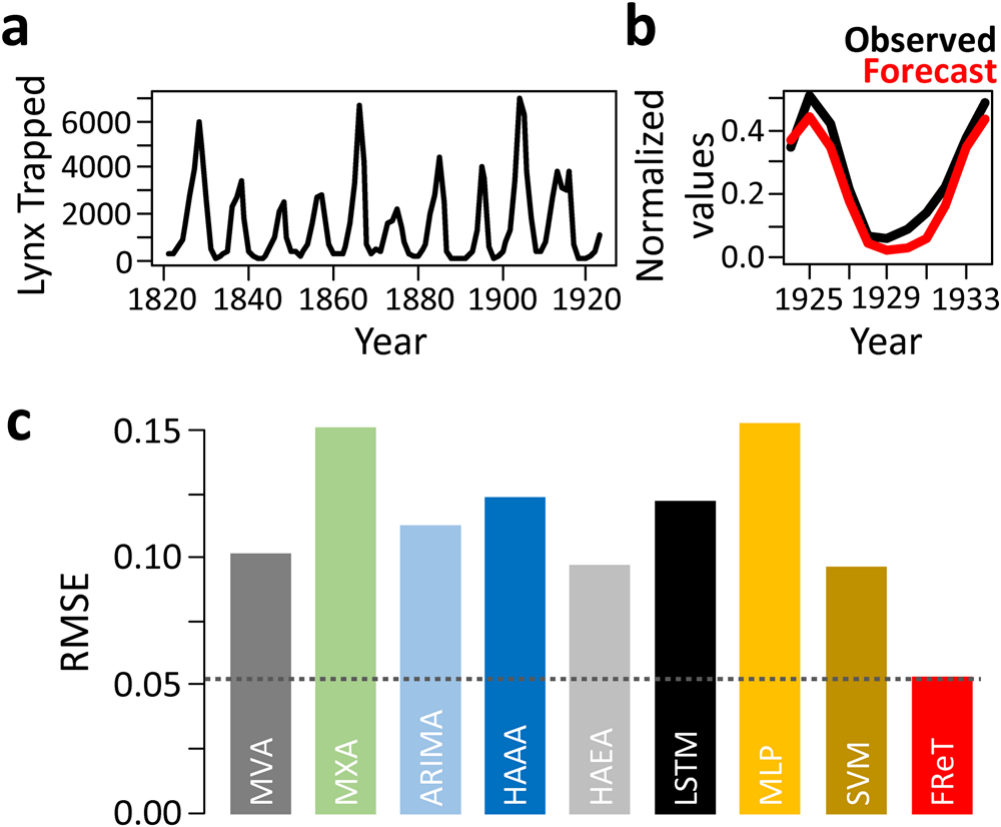
Annual record of Canadian lynx trapped in the Mackenzie River district of North-West Canada. **a** Annual lynx trapped from 1821 to 1923 illustrating persistent oscillations and substantial irregularities in amplitude which shows no systematic trend. **b** Forecasted data from 1924 to 1934. Black denotes real data and red denotes Forecasting through Recurrent Topology (FReT) forecast of unseen data. **c** Root-mean-square-error (RMSE) of a multi-step-ahead FReT forecast compared to several recently and independently benchmarked models: Maximum Visibility Approach (MVA), Mao-Xiao Approach (MXA), Autoregressive Integrated Moving Average (ARIMA), Hybrid Additive ARIMA-ANN (HAAA), Hybrid Additive ETS-ANN (HAEA), Long Short-Term Memory (LSTM), Multilayer Perceptron (MLP), and Support Vector Machine (SVM). Similar data normalization and 11-year forecast horizon was used. Source: Moreira et al. 2022.

Finally, the Canadian lynx dataset is a well-known dataset that has long been associated with time-series analysis^4,18^. It has been recently and independently benchmarked using a variety of forecasting techniques including neural network-based models^41^. The dataset consists of the annual Canadian lynx trapped in the Mackenzie River district of North-West Canada for the period 1821-1934 that reflect fluctuations in the size of the lynx population^4,18^. As shown in Fig. 7a, the most striking feature of the plot is the presence of persistent oscillations with a period of about ten years. However, there are substantial irregularities in amplitude, which although familiar to biologists, shows no systematic trend^4^. Using similar data normalization and forecast horizon, a single multi-step-ahead forecast (Fig. 7b) was able to outperform these independently benchmarked models (Fig. 7c and Supplementary Table 1). In fact, the RMSE of FReT was almost half that of the best-performing models (Fig. 7c).

## Discussion

Forecasting time-series data has often relied on highly parameterized or black-box models that bring with them a variety of drawbacks. The performance of these models highly depends on their architecture and chosen hyperparameters^28^. Appropriate design of these models is, therefore, critical. Even with the appropriate design, however, we are not guaranteed better performance^15,20^. In fact, despite its simplicity, the ability of FReT to make comparable or even better forecast predictions than parameterized models further highlights the misconception that more complex models are more accurate, and thus complicated black-box models are necessary for top predictive performance^24^.

It has traditionally been thought that techniques that exhibit top performance are difficult to explain/visualize. Neural networks, for instance, can have layered architectures that effectively model complex data features but are hard to explain using formal logic^42^. On the other hand, linear methods are easy to explain because they can be described using linear equations. However, data in the real world are often nonlinear, so linear methods often do not perform as well^42,43^. Consequently, there has been a surge of interest in recent years in studying how complex models work, and how to provide formal guarantees for these models and their predictions^19,42^.

It has been proposed that models should exhibit four key elements. First, they should be Explainable: The inner workings of produced predictive models should be interpretable, and the user should be able to query the rationale behind the predictions. Second, they should be Verifiable: The compliance of the produced models with respect to user specifications should be formally verifiable. Third, they should be Interactable: Users should be able to guide the learning phase of predictive models so that the models conform with given specifications. Finally, the models should also be Efficient: Models should only consume reasonable resources to complete learning and prediction tasks^42^. All four of these elements depend on model complexity. That is, with increasing model complexity, efficiency and explainability tend to decrease, while the need for verifiability and interactability tend to increase. FReT offers a simple approach for time-series forecasting that lacks model complexity, avoiding critical user specifications and a real need for verifiability and interactability. Users are also able to both visualize and query the rationale behind the predictions (e.g., Supplementary Movie 1), and the lack of optimization and tuning procedures for specifying hyperparameters in high-dimensional parameter space can improve computational efficiency. Thus, the development of algorithms such as FReT that do not require optimization and tuning procedures represent an important step towards prioritizing computationally efficient algorithms^21,22^.

FReT also has a unique property in that the identification of topological archetypes may also be used for cross-validation across multidimensional systems. For example, when individual archetypes converge on a similar forecasted trajectory across dimensions (i.e., the greater number of archetypes from different dimensions that forecast similar events), we can be more confident that those events are likely to occur. This feature may be particularly advantageous when model output is highly sensitive to the chosen input hyperparameters, or under more practical situations when we truly do not know the ground truth values. That is because it would be difficult to trust the predictions of these models without testing how much they depend on their estimated input hyperparameters.

It is important to note that FReT forecasts are based on the original data. Therefore, forecasts are related to the original scale. This eliminates any potential transformation bias when converting back to the original scale in situations when transformations are needed for model fitting^44^. The scaling/normalizing of data in this study was only done to compare to previous data. A limitation of this approach, on the other hand, is its dependence on recurring patterns. The requirement that the system must have experienced the forecasted state (or a closely approximated state) at some prior point in time may therefore require longer duration temporal sequences for more accurate forecasting. This may be particularly relevant for more complex signals. Nevertheless, common ground between many different types of time-series data resides in their shared property of embedded patterns^45^.

Our data indicate that FReT can provide accurate forecasts while offering a distinct computational advantage compared to highly parameterized models such as artificial neural networks. FReT is also able to do this while avoiding the drawbacks associated with artificial neural networks including vulnerability to overfitting, random matrix initializations, and the need for optimization and tuning techniques. In fact, application of learning through the proposed FReT framework may offer a simple approach for continuous model updating capabilities. For instance, gait kinematic trajectory prediction using wearable sensors can be used to solve numerous problems facing robotic lower-limb prosthesis/orthosis^1^. However, a limiting factor for the implementation of accurate gait forecasting in the design of next-generation intelligent devices is the inability of modern forecasting models to support continuous model updating. Continuous model updating would enable adaptive learning to continuously incorporate user-specific^46,47^ and current dynamic signal information to increase device functionality and user safety^31–34^, while avoiding prediction errors when pretrained optimized models are used under conditions that have not been included in the initial training process^48,49^. This is particularly relevant for gait which is dynamically modulated to adjust for differing environmental conditions, and to meet the needs of ever-changing motor demands^50^. Thus, unlike FReT, the complexity of modern prediction models poses a tangible barrier as these models can consume extended durations for hyperparameter optimization (Supplementary Fig. 3), negating the potential for continuous model updating.

In conclusion, this paper introduces FReT, a prediction algorithm based on learning recurrent patterns in a series’ local topology for forecasting time-series data. The proposed method was tested with a variety of datasets and was compared to several parameterized and benchmarked models. With no need for computationally costly hyperparameter optimization procedures in high-dimensional parameter space, FReT offers an attractive alternative to complex models to reduce computational load and power consumption constraints.

## Methods

The main goal of time-series prediction is to collect and analyze past time-series observations to enable the development of a model that can describe the behaviour of the relevant system^28^. SETAR models have a long history of modelling time-series observations in a variety of data types^3,6,7,17,18,51–53^. They are nonlinear statistical models that have been shown to be comparable or better than many other forecasting models including some neural network-based models on real-world data^3,6,7,17,51,52^. SETAR models also have the advantage of capturing nonlinear phenomenon that cannot be captured by linear models, thus representing a commonly used classical model for forecasting time-series data^3,6,7,17,51–53^.

The most common approach for SETAR modelling is the 2-regime SETAR (2, *p*_1_, *p*_2_) model where *p*_1_ and *p*_2_ represent the autoregressive orders of the two sub-models. This model assumes that a threshold variable is chosen to be the lagged value of the time-series, and thus is linear within a regime, but is able to move between regimes as the process crosses the threshold^7,17,18^. This type of model has had success with respect to numerous types of forecasting problems including macroeconomic and biological data^3,6,7,17,51–53^. However, SETAR model autoregressive orders and the delay value are generally not known, and therefore need to be determined and chosen correctly^6^.

In recent years, machine-learning methods, including NNET models have attracted increasingly more attention with respect to time-series forecasting. These models have been widely used and compared to various traditional time-series models as they represent an adaptable computing framework that can be used for modelling a broad range of time-series data^6,41,43^. It is therefore not surprising that NNET is becoming one of the most popular machine-learning methods for forecasting time-series data^6,43^. The most widely used and often preferred model when building a NNET for modelling and forecasting time-series data is a NNET with a Multilayer Perceptron architecture given its computational efficiency and efficacy^6,18,43,54,55^ and its ability to be extended to deep learning^1^. There are two critical hyperparameters that need to be chosen, the embedding dimension and the number of hidden units^18^. For deep learning, there is a third critical hyperparameter that also needs to be selected; the number of hidden layers. The choice of the value of hidden units depends on the data, and therefore must be selected appropriately. Perhaps the most crucial value that needs to be chosen is the embedding dimension as the determination of the autocorrelation structure of the time-series depends on this^6^. However, there is no general rule that can be followed to select the value of embedding dimension. Therefore, iterative trials are often conducted to select an optimal value of hidden units, embedding dimension, and number of hidden layers (for deep learning), after which the network is ready for training^1,6,18^.

Many parameterized prediction models, including SETAR and artificial neural networks, are often limited in that performance of these models highly depends on the chosen hyperparameters such as embedding dimension, delay value, or model architecture. These types of models can also require tuning and optimization in high-dimensional parameter space which can have an impact on model selection, performance, and system-level constraints such as cost, computational time, and budget^23^. Thus, the motivation for this work was to overcome these drawbacks and develop a simple, yet effective general-purpose algorithm with no free parameters, hyperparameter tuning, or critical model assumptions. The algorithm is based on identifying recurrent topological structures that can be used to forecast upcoming dynamic changes and is introduced next.

### Forecasting through Recurrent Topology (FReT)

As dynamic systems can exhibit topological structures that may allow predictions of the system’s time evolution^11,56^, an algorithm that can reveal unique topological patterning in the form of memory traces embedded in a signal may offer an approach for dynamical system forecasting. Local topological recurrence analysis is an analytical method for revealing emergent recurring patterns in a signal’s surface topology^40^. It has been shown to be capable of outperforming neural network-based models in revealing digital biomarkers in time-series data^40^, and may therefore offer a computational tool to decode topological events that may reflect a system’s upcoming dynamic changes. However, to be able to forecast based on recurring local topological patterning, we would first need to find prior states that share overlapping recurring patterns with respect to the system’s current state. Importantly, we need to be able to do this using a 1-D time-series. This would eliminate the need for time-delay embedding hyperparameters and the uncertainty associated with their estimation. If these overlapping recurring patterns, or archetypes, can be identified, they could be used for decoding complex system behaviours relevant to a dynamic system’s current state, and thus its expected future behaviour.

For instance, consider a data sequence where ${{{{{\rm{x}}}}}}$ represents a 1-D time-series vector with ${x}_{n}$ indexing the system’s current state:

1 $${{{{{\rm{x}}}}}}=({x}_{1},{x}_{2},{x}_{3},\ldots ,{x}_{n})$$

With local topology, this 1-D signal is transformed into a local 3 × 3 neighbourhood topological map based on the signal’s distance matrix:

2 $${T}_{{ij}}=\left(\begin{array}{ccc}{D}_{i-1j-1} & {D}_{i-1j} & {D}_{i-1j+1}\\ {D}_{{ij}-1} & {D}_{{ij}} & {D}_{{ij}+1}\\ {D}_{i+1j-1} & {D}_{i+1j} & {D}_{i+1j+1}\end{array}\right)$$

where ${D}_{{ij}}$ represent the elements of the $n\times n$ Euclidean distance matrix. This approach represents a general-purpose algorithm that works directly on 1-D signals where the 3 × 3 neighbourhood represents a local point-pair’s closest surrounding neighbours^40^. While different neighbourhood sizes can be used, a 3 × 3 neighbourhood provides maximal resolution. The signal’s local topological features are then captured by different inequality patterning around the 3 × 3 neighbourhood when computed for all ${T}_{{ij}}$ by constructing the matrix (${T{{\hbox{'}}}}$) that represents an 8-bit binary code for each point-pair’s local neighbourhood:

3 $${({T}_{{ij}}^{{\prime} })}_{8}=\mathop{\sum }\limits_{q=1}^{8}s({g}_{q}-{g}_{0}){2}^{q-1}{{{{{\rm{;}}}}}}s\left(x\right)=\left\{\begin{array}{c}0,x \, < \, 0\\ 1,x\ge 0\end{array}\right.$$

Here *g*_*0*_ represents $({D}_{{ij}})$ and ${g}_{q}=\{{g}_{1},\ldots ,{g}_{8}\}$ are its eight-connected neighbours^40^. Each neighbour that is larger or equal to *g*_*0*_ is set to 1, otherwise 0. A binary code is created by moving around the central point *g*_*0*_ where a single integer value is calculated based on the sum of the binary code elements (0 or 1) multiplied by the eight 2^p^ positional weights. This represents 8-bit binary coding where there are 2^8^ (256) different possible integer values, ranging from 0 to 255, that are sensitive to graded changes in surface curvature of a dynamic signal^40^. The range is then divided into sextiles to create 6 integer bins that are flattened into a 2-D matrix (Fig. 1) where this 2-D $m\times m$ matrix ($m=n-2$) can be thought of as a set of integer row vectors (${\mathop{x}\limits^{ \rightharpoonup }}_{i}$) with the last row vector (${\mathop{x}\limits^{ \rightharpoonup }}_{m}$) representing the system’s current state. We can then determine the similarity of ${\mathop{x}\limits^{ \rightharpoonup }}_{m}$ to all other prior states, ${\mathop{x}\limits^{ \rightharpoonup }}_{i}$:

4 $${\mathop{x}\limits^{ \rightharpoonup }}_{i}={x}_{i}^{1},{x}_{i}^{2},\ldots ,{x}_{i}^{m}$$

using a simple Boolean logic-based similarity metric ${S}_{{im}}$:

5 $${S}_{{im}}= \,	\frac{{\sum }_{m=1}^{m}[a\left({x}_{i}^{1}-{x}_{m}^{1}\right),a\left({x}_{i}^{2}-{x}_{m}^{2}\right),\ldots ,a\left({x}_{i}^{m}-{x}_{m}^{m}\right)]}{m}; \\ a\left(x\right)= \,	\left\{\begin{array}{c}1({{{{{\rm{True}}}}}}),x=0\\ 0({{{{{\rm{False}}}}}})\hfill\end{array}\right.$$

Here, each element-wise difference in row vectors ${\mathop{x}\limits^{ \rightharpoonup }}_{m}$ and ${\mathop{x}\limits^{ \rightharpoonup }}_{i}$ are computed, generating a 1 (True) if their difference equals zero, otherwise 0 (False). This ${S}_{{im}}$ similarity metric, which differentially weights the importance of each part of the input data, can therefore range from 0 to 1, with values approaching 1 being weighted stronger. This produces a 1-D weight vector with respect to the system’s current state where higher values represent topological sequences that more closely align with the system’s current state (e.g., Supplementary Movie 1). The operations associated with Eqs. 2 and 3 are therefore important as they enable local contextual information to distinguish between signal data points with similar scalar values. In other words, they help reveal archetypes based on topological sequence patterning rather than the closest points in state space which requires the assumption that future behaviour varies smoothy.

We can now define a set of ${S}_{{im}}$ threshold values ranging from around 0.6 to 1.0 with which to maximize to find ${\mathop{s}\limits^{ \rightharpoonup }}_{i}$, a row vector from the set of all ${\mathop{x}\limits^{ \rightharpoonup }}_{i}$ that is highly similar to the local topology state changes of the system’s current state, ${\mathop{x}\limits^{ \rightharpoonup }}_{m}$:

6 $$\{{\mathop{s}\limits^{ \rightharpoonup }}_{i}\subseteq {{\mathbb{Z}}}^{1\times m}\mid{{{{{\mathcal{P}}}}}}\left({\mathop{s}\limits^{ \rightharpoonup }}_{i}\approx {\mathop{x}\limits^{ \rightharpoonup }}_{m}\right),{{{{{\rm{with}}}}}}\,{S}_{{im}}\,{{{{{\rm{threshold\; maximization}}}}}}\}$$

Thus, topological archetype detection effectively reduces to a simple maximization problem where the row index of ${\mathop{s}\limits^{ \rightharpoonup }}_{i}$ + 3 (to account for the $m\times m$ matrix dimensions and a forecast starting at $n$ + 1 in the future) equals the index of the encoded archetype in ${{{{{\rm{x}}}}}}$ (Eq. 1). In principle, threshold maximization will find the archetype (row vector) with greatest similarity. However, for more robust point estimates, we can subject the maximization to the constraint: a minimum of ≥3 ${\mathop{s}\limits^{ \rightharpoonup }}_{i}$. This was used in this study unless otherwise stated. Under this condition, the element-wise average of the signal trajectory extending out from the encoded regions are used to model the forecast, where the standard error can be used as a metric of uncertainty. For nonstationary long-run mean data, the encoded signal is first centred before the element-wise average is computed, and the modelled forecast remapped to the current state by adding the difference between the last data point of the series and the first point of the centred forecast.

### Datasets

For the initial illustrative examples, a simple sine wave was constructed by a sequence of 300 points ranging from 0.1 to 30 with an interval of 0.1. For the string of text, a well-known Dr. Seuss book excerpt that has been used for time-series analysis was used^57^. For more complex dynamic systems, the Rössler (*a* = 0.38, *b* = 0.4, *c* = 4.82, and ∆*t* = 0.1) and Lorenz (*r* = 28, *σ* = 10, $\beta$ = 8/3, and ∆*t* = 0.03) attractor systems were used with initial parameters based on previous values^10,25^. Every second data point was used for analysis of these time-series, so the same duration was covered, but with only half the data points. The publicly available embedded versions of the Mackey-Glass time-series were also used in this study^27^.

Both the population and macroeconomic datasets used in this study are available in R^4,18,29^. The lynx data consists of the annual record of the number of Canadian lynx trapped in the Mackenzie River district of North-West Canada for the period 1821-1934^4,18^. The macroeconomic datasets used here correspond to the U.S.-Canadian dollar exchange rate from 1973 to 1999^18,29^, and the month U.S. unemployment rate from January 1948 to March 2004^18,30^.

Gait data were analyzed from a heterogeneous sample of five young to middle-aged adults without gait impairment^40^ using a single wearable sensor^58^. The sensor system is based on using motion processor data consisting of a 3-axis Micro-Electro-Mechanical Systems (MEMS)-based gyroscope and a 3-axis accelerometer. The system’s firmware uses fusion codes for automatic gravity calibrations and real-time angle output (pitch, roll, and yaw). The associated software application utilizes sensor output for gait biometric calculations in real-time while recording gait-cycle dynamics and controlling for angular excursion and drift^58–60^. The sensor is attached to the leg just above the patellofemoral joint line through the use of a high-performance thigh band which is the optimal location for this sensor system^58–60^.

### Data analysis

In addition to recent benchmark data generated in the literature, SETAR, NNET, and D-NNET models were also used for FReT comparative analysis. For the SETAR models, different forecasting methods were used for testing (Naïve, Bootstrap resampling, Block-bootstrap resampling, and Monte-Carlo resampling)^18^. For macroeconomic model building, a logarithmic transformation (log10) was first applied to the data as commonly done^61^. Specific model details and network architectures are noted when presented. For FReT, data were log-transformed after forecasting to enable comparison to SETAR, NNET, and D-NNET models.

### Supplementary information

Supplementary Information

Description of Additional Supplementary Files

Supplementary Movie 1

## Data Availability

The datasets used in this study can be found at https://github.com/tgchomia/ts.

## Code availability

R software (R Core Team. R: A language and environment for statistical computing. R Foundation for Statistical Computing, Vienna, Austria) and associated source code and packages (https://www.R-project.org/) are publicly available. Data and FReT example with code can be found at https://github.com/tgchomia/ts. NNET and SETAR code is available in R package tsDyn^18^. D-NNET code is available in R package nnfor^62^.
