# Supplementary material for: Time-series forecasting through recurrent topology
Source: Commun Eng. 2024 Jan 9;3:9. doi: 10.1038/s44172-023-00142-8 (PMC10956040; doi:10.1038/s44172-023-00142-8)
Supplement: Supplementary file 1 — Supplementary Information [file 44172_2023_142_MOESM1_ESM.pdf]

Supplementary Figures

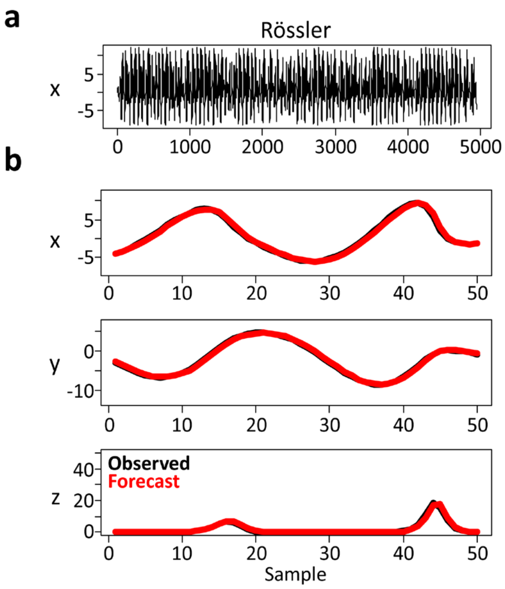

Supplementary Figure 1. Multidimensional forecasting from a single dimension in the Rössler attractor system

a: x-dimension of the Rössler system that was used for topological archetype identification. b: The forecasted trajectories in all x, y, and z dimensions from a single x-dimension archetype (black: unseen observed data, red: forecast).

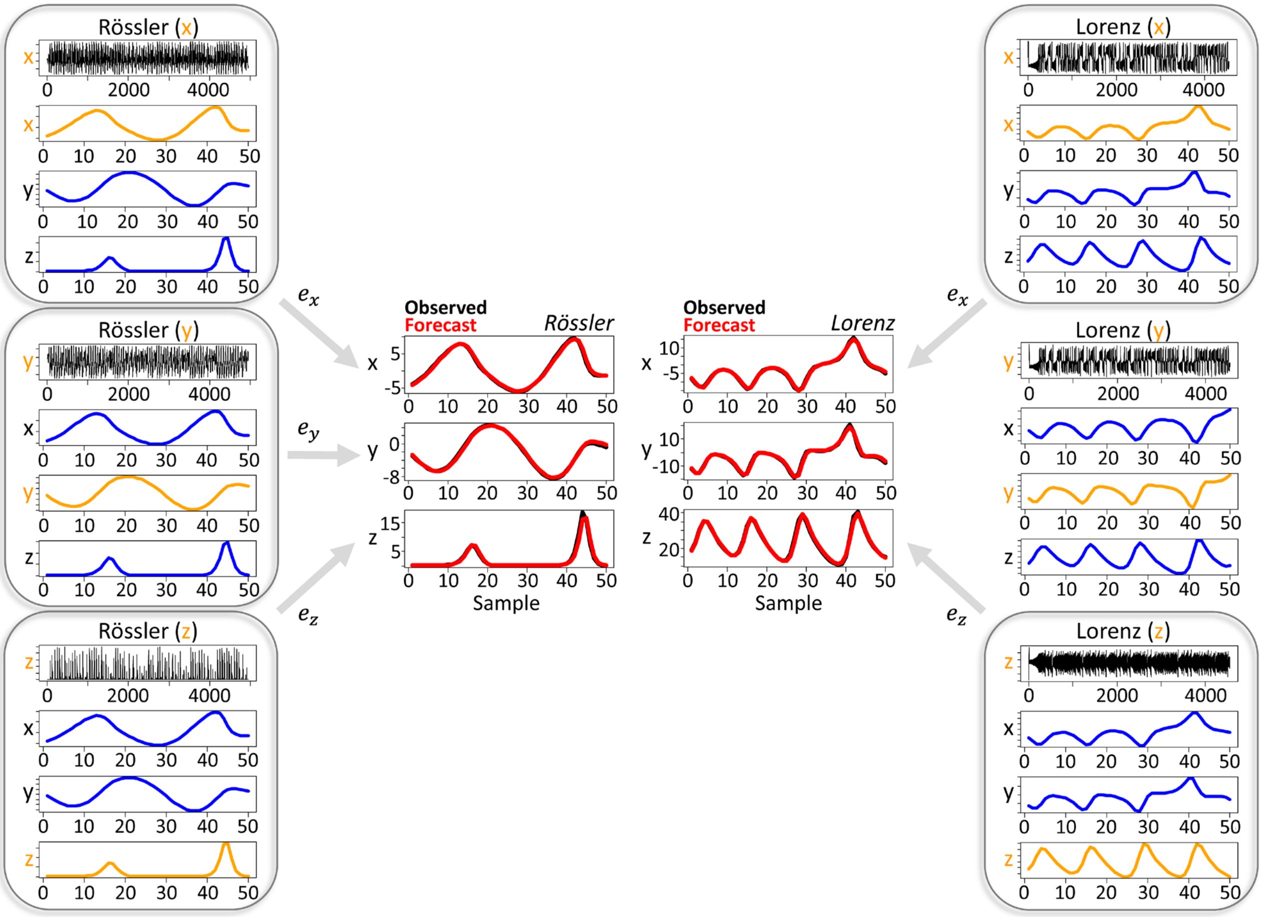

Supplementary Figure 2. Archetype cross-validation across multidimensions

Exemplar archetypes can also be used for cross-validation across multidimensional systems (e_i) in which individual archetypes that converge on a similar forecast trajectory across dimensions have the added benefit of providing a measure of confidence in the predictions. Illustrated here, Rössler (left) and Lorenz (right) systems where a single dimensional archetype (orange trace) was used to forecast across all dimensions (orange and blue traces) assuming ground truth is unknown. For the Rössler system, all archetypes (e_x, e_y, e_z) predict similar trajectories, while for the Lorenz system, archetypes e_x and e_z predict similar forecasted trajectories across the entire forecast length. Using the mean of the encoded archetypes with convergent trajectories, the forecasted dynamics are shown in red with the ground truth shown in black.

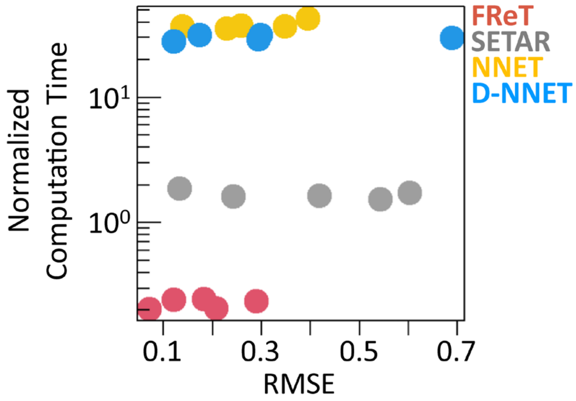

Supplementary Figure 3. Gait kinematic forecasting with different models

Subject-wise forecast accuracy (RMSE) for different models. Normalized computational time (plotted on a log scale) is relative to forecast horizon duration. Values $>10^0$ reflect processing times that are longer than the forecast horizon. NNET (yellow) and D-NNET (blue) forecasts were based on an optimized neural network architecture for each individual. Model selection for NNET forecasting was based on optimizing across 20 embedding dimensions and 20 hidden units, while optimizing D-NNET was based on optimizing across 5 hidden units, but 3 layers deep. Model selection for SETAR (grey) forecasting was based on optimizing across 6 embedding dimensions and 6 threshold delays. FReT is shown in red. FReT, Forecasting through Recurrent Topology; SETAR, Self-Exciting-Threshold Nonlinear Autoregressive Model; Artificial Neural Network (NNET); Deep-NNET (D-NNET).

Supplementary Table 1. Forecasting through Recurrent Topology compared to recent benchmarks on the lynx time-series

Model	MAE	MAPE
MVA	0.0714	46.72
MXA	0.1393	64.92
ARIMA	0.0877	32.88
HAAA	0.1115	54.18
HAEA	0.0725	43.07
LSTM	0.1053	155.94
MLP	0.1267	60.78
SVM	0.0828	39.48
FReT	0.0495	29.19

Maximum Visibility Approach (MVA), Mao-Xiao Approach (MXA), Autoregressive Integrated Moving Average (ARIMA), Hybrid Additive ARIMA-ANN (HAAA), Hybrid Additive ETS-ANN (HAEA), Long Short-Term Memory (LSTM), Multilayer Perceptron (MLP), Support Vector Machine (SVM), and Forecasting through Recurrent Topology (FReT). Similar data normalization and forecast horizon was used. Source: Moreira et al., 2022.
