# Supplementary material for: Time-series forecasting through recurrent topology
Source: Commun Eng. 2024 Jan 9;3:9. doi: 10.1038/s44172-023-00142-8 (PMC10956040; doi:10.1038/s44172-023-00142-8)
Supplement: Supplementary file 2 — Description of Additional Supplementary Files [file 44172_2023_142_MOESM2_ESM.pdf]

Description of Additional Supplementary Files

File name: Supplementary Movie 1

Description: Video illustrating how data point archetypes can exist in a dynamic system’s signal output and encode system behaviours about the system’s current state. Spikes reveal open-reading-frames embedded in the signal that represent an embodied model about the system’s upcoming behaviour.
